# Supplementary material for: Test-retest reliability of the six-minute walking distance measurements using FeetMe insoles by completely unassisted healthy adults in their homes
Source: PLOS Digit Health. 2023 Nov 22;2(11):e0000262. doi: 10.1371/journal.pdig.0000262 (PMC10664940; doi:10.1371/journal.pdig.0000262)

Haleh Ayatollahi, PhD
Section Editor
PLOS Digital Health

August 16th, 2023.

Dear Dr. Ayatollahi,

We would like to express our gratitude for the invitation to submit a revised version of our manuscript, entitled "Excellent test-retest reliability of the six-minute walking distance measured by FeetMe insoles during tests conducted with a one-week interval by completely unassisted healthy adults in their homes". We sincerely appreciate the valuable time and effort you, as well as each of the reviewers, have dedicated to providing insightful feedback on how to enhance our paper. It is with great pleasure that we present our resubmitted article for further consideration. We have diligently incorporated changes that align with the detailed suggestions you generously provided. We hope that our edits, as well as the responses we provide below, effectively address all the issues and concerns highlighted by you and the reviewers.

To aid your review of our revisions, we have prepared a point-by-point response to the questions and comments outlined in your letter dated June 22th, 2023.

EDITOR REQUESTS:

We express our appreciation to the editorial team for their valuable remarks, which we have carefully considered, as explained in the following section.

1. Please provide separate figure files in .tif or .eps format only and remove any figures embedded in your manuscript file. Please also ensure that all files are under our size limit of 10MB.

   Response: we removed the figures from the manuscript and converted them to .tiff format using *PACE* tool, as suggested on your website.

2. We noticed that you used unpublished data in the manuscript. We do not allow these references, as the PLOS data access policy requires that all data be either published with the manuscript or made available in a publicly accessible database. Please amend the supplementary material to include the referenced data or remove the references.

   Response: we updated the manuscript with reference to the publicly accessible article, which is also currently in peer review in another journal. We would like to re-update the reference once the article is published in the journal.

3. We do not publish any copyright or trademark symbols that usually accompany proprietary names, eg ©, ®, ™ (e.g. next to drug or reagent names). Please remove all instances of trademark/copyright symbols throughout the text, including ®.

   Response: all symbols were removed from the text, as per your request.

4. Figure 1 contains screenshots. We are not permitted to publish these under our CC-BY 4.0 license; websites are usually intellectual property and are copyrighted. This includes peripheral graphics of the web browser such as the buttons. We ask that you please remove or replace it.

Response: we removed the graphics following your request

5. In the online submission form, you indicated that "Data available on request due to restrictions e.g. privacy or ethical". All PLOS journals now require all data underlying the findings described in their manuscript to be freely available to other researchers, either:
   1. In a public repository
   2. Within the manuscript itself, or
   3. Uploaded as supplementary information.

   This policy applies to all data except where public deposition would breach compliance with the protocol approved by your research ethics board. If your data cannot be made publicly available for ethical or legal reasons (e.g., public availability would compromise patient privacy), please explain your reasons by return email and your exemption request will be escalated to the editor for approval. Your exemption request will be handled independently and will not hold up the peer review process, but will need to be resolved should your manuscript be accepted for publication. One of the Editorial team will then be in touch if there are any issues.

   Response: the data is now available in a public repository: https://doi.org/10.5281/zenodo.8252988.

REVIEWER 1 COMMENTS:

We express our gratitude to the reviewer for dedicating their time and providing us with beneficial feedback. Below, we outline our responses to the comments raised by the reviewer.

1. While the conflict of interest have been disclosed, a majority of the authors are/have been related to the company providing the device tested, in addition of the financial support provided by the FeetMe company. This requires a measure of caution when drawing conclusions and should be further highlighted within the text, preferably in a dedicated limitations sections.

   Response: we thank the reviewer for this relevant suggestion. To address this concern the phrase "*Besides, some of the authors of this work were affiliated with FeetMe company. Future studies by an independent group of researchers are therefore required to replicate these results in a larger sample population, including both healthy volunteers and those with gait anomalies, with in-hospital tests conducted at multiple centers and in multiple countries. In addition, the learning effect between repeated 6MWTs at home should be further studied and compared to that in hospital settings.*" was added to the newly created '*Study limitations*' section (lines 291-295).

2. On a related note, some of the literature used to support the potentials of the solution (14, 15) included authors who were employees of the FeetMe company, hence indicating need for caution regarding conflicting interests. Deriving such potentials from independent studies would add more value to the claims.

   Response: we thank the reviewer for this relevant suggestion. In fact, two out of four cited works were performed by independent groups. To address this concern, independent studies were put forward and an additional independent study was cited. However, we still cite the works that raised reviewer's concerns. Independent researchers have participated in these

studies and their results were published in renown peer-reviewed journals. We think they have merit to be cited here. The manuscript was modified as follows: "*These insoles were designed to assess many gait parameters, including stride length, velocity, stance, swing, step, single and double support durations, and cadence and their reliability have already been prov-en by independent studies. Test-retest reliability of gait parameters was confirmed in older adults [14] and the insoles were found to be as reliable as the GAITRite clinical walkway system for walking tests con-ducted in clinical settings in patients with multiple sclerosis [15] and Parkinson's disease [16]. Other studies in patients with post-stroke [17] and in healthy volunteers [18] came to similar conclusion.*" (lines 87-93)

3. Some clarifications on the procedures and results would also be beneficial to add to the body of literature. For example, in the Intervention section, the length of the training of the participants could have been specified. Reasons for why some patients missed taking the required test and the relevant technical issues would also be important to share.

   Response: we thank the reviewer for this relevant suggestion, the training duration was added (line 138). Regarding the reasons why some participants missed taking the test, we didn't have the opportunity to query all of them, but we tried to add more information to the manuscript (lines 183-188). In many cases, the data completeness was checked after the last visit of the participant, so if an issue was found, it was not always possible to communicate with the participant regarding the given issue.

4. As the use of a companion smartphone application has been made, its compliance to relevant privacy policies should be specified. The field of digital health is not focused only on the technological component but also integrates the relevant ethical, social and regulatory concerns. As the FeetMe smart insoles is a commercial product, acknowledging its compliance to relevant policies would be a recommendation for scientific publication.

   Response: we thank the reviewer for this relevant suggestion. To address this concern it was explicitly stated, that the FeetMe Evaluation smartphone application is Class Im CE(93/42/EC) and Class I FDA 510(k) exempt medical device (line 124).

5. While the authors compared their findings with other research in gait studies, they did not do so with other studies that analysed other smart insoles for similar purposes. There have been a number of such studies in the past 5 years or so which the authors could compare their findings to in order to provide a more objective analysis of their findings.

   Response: we thank the reviewer for this suggestion. We agree with the reviewer that many publications related to smart insoles do exist. However, the data reported in this work relates to the test-retest reliability of the 6 minutes walking distance measurements. Our findings can only be compared to the same type of measurements. Search on PubMed with key words "6-minutes walk test smart insole" and "6MWT smart insole" gave no results. Hence, we were not able to find any such studies that used smart insoles and to which we can compare our findings. In addition, in our opinion, the problem we are trying to solve is not linked to a particular kind of solution, be it an insole or a mobile app as soon as a reliable remote assessment of patient's performance is possible. That is why we compare between such solutions and not between similar types of technologies.

6. It could also be recommended for the authors to use more cautious language when deriving recommendations in the discussion and conclusions. This is because the participants were healthy and the generalisability of the findings can be challenging to extrapolate to patients

with gait disturbances. Further limiting the generalisability is that only 4 interventions took place for each participant. Thus, it could also be recommended to rephrase the study title as a feasibility assessment or pilot to be more indicative of the study scope.

Response: we understand the reviewer's concern, yet we find that rather cautious language is already used in our manuscript. As mentioned in the '*Introduction*', the solution was already validated for measuring the gait in healthy adults, elderly, patients with multiple sclerosis, patients with Parkinson's and patients post stroke ([14-18]), which hints at a possible extrapolation of our result to patients with gait disturbances. On the other hand, in the '*Study limitations*' section, we do clearly mention this fact as a limitation: "*this study <…> involved healthy volunteers rather than patients with pathological gait. <…> Future studies <…> are therefore required*" (lines 289-292). In any case, to comply with the reviewer's request, we modified the language to a more cautions one in the '*Discussion*' section as follows: "*Thus, at-home self-administered 6MWTs using the FeetMe technology were found to be at least, if not more, reliable than rater assessments conducted in a hospital setting, providing evidence that the FeetMe insoles could deliver an easy-to-use, reliable, and accurate solution allowing patients to conduct 6MWTs at home.*" (lines 231-234). We also edited the '*Conclusion*' section quite significantly (lines 297-309).

REVIEWER 2 COMMENTS:

The reviewer's time and valuable feedback are greatly appreciated, and we extend our thanks for their efforts. Our comprehensive replies to the reviewer's comments are elaborated upon below.

1. In line 58, please specify the type of bias and explicitly state how it affects measurement.

   Response: in this particular case, by bias we mean systematic error. We put it explicitly in the text to make it clear (line 163). Bias or systematic error is a very common term, we don't think that after this precision, we need to further elaborate on this term in the manuscript.

2. The language used in line 81/82 is misleading, given that the authors are reporting unpublished observations. Please provide more details on this study and explicitly state in text that these results require additional testing and/or peer review.
   Response: we thank the reviewer for pointing this out. The observations are now published and are under peer review in another journal. We now explicitly cite the article in the manuscript and mention that it is under peer review (line 95).
3. In line 84, the authors state that they are assessing reliability and repeatability of the FeetMe. In later sections it seems that they are only assessing test-retest reliability (which can also be called repeatability). Is there another form of reliability being assessed? Additionally, in line 135 the authors state that their primary outcome was to compare the test-retest reliability of at home vs by the rater in the hospital. Please use consistent language and goals throughout the manuscript.

   Response: we thank the reviewer for pointing out this lack of clarity in our manuscript. Indeed, only test-retest type of reliability was investigated. We modified the paragraph to take reviewer's remark into account: "*The aim of the current study was to probe the test-retest reliability of FeetMe insoles in estimating the 6MWDs in a home environment. This was accomplished by assessing the repeatability of the 6MWTs completed by healthy volunteers in their homes with a one-week interval between the tests*" (lines 95-98). We also clarified the link

between at home vs by the rater reliability in the '*Outcomes*' section: "*The main outcome was the test-retest reliability of the FeetMe 6MWD measurements from tests performed by the participants at home without any assistance. The test-retest reliability of the 6MWD measurements made by the rater in hospital and by FeetMe in hospital in the same population was used as a reference level of repeatability*" (lines 156-159).

4. The hypothesis for test-retest reliability, including a targeted kappa score is not provided. This is concerning as it prevents the reader from understanding what the initial goal was and whether or not it was met.

   Response: we kindly disagree with the reviewer. The goal is to assess the test-retest reliability of the FeetMe device at home and to compare it to the degree of repeatability of the evaluation done by the rater, which is the way the 6MWD is evaluated today in clinical practice. The goal is thus to see whether the device has a sufficient level of repeatability to replace a well-established method of performing the test. We made this clearer in the new revision, so we believe that there are no more barriers for understanding the initial goal and whether it was reached or not. Regarding the kappa score, as it is explained further in the letter this approach is not suitable for the analysis of the distances.

5. Please provide details on how participants were recruited. Were all participants recruited through one clinic? This information may help readers understand the context of the study and its generalizability.

   Response: as stated in the manuscript, it is a single-center study (line 101), meaning that all participants were recruited through one clinic. This fact is also mentioned in the '*Study limitations*' section as a study limitation (line 291). Following the reviewer's request, we added a sentence describing the recruitment process: "*The participants were recruited using study posters displayed in the relevant areas (e.g. universities for the younger age groups, typical associations for older age groups)*" (lines 115-116).

6. Information on who rated the 6MWT would be useful.

   Response: we thank the reviewer for this suggestion. We added the following information to the manuscript: "*In hospital, the participants were evaluated by a rater (a medical doctor or an engineer) trained to administer the 6MWT according to the study protocol*" (lines 143-144).

7. Please provide additional information on if soles were adapted for different feet types/sizes. Additionally, if the soles were ill-fitting, would this affect the results provided? Please describe.

   Response: we thank the reviewer for this suggestion, however it is mentioned in the manuscript, that the insoles are adapted for feet/shoe sizes between 35 and 46 (EU) (line 121). To address the reviewer's concern, we added the following phrase to the '*Intervention*' section: "*The investigator also helped selecting the insoles size and insured that they fit well participants' shoes*" (lines 140-141).

8. The age range included in this study was quite wide. Were there any subgroup analysis made, or any noted differences included across different age ranges?

   Response: we thank the reviewer for this relevant question. No subgroup analysis was made, as the per-subgroup number of participants is rather low, at least for some subgroups. We will perform such an analysis in the subsequent work using this study data but keep it out of the scope of this manuscript.

9. I appreciated the use of several statistical tests. For test-retest reliability, kappa statistics to estimate the proportion of observed agreement to the proportion of agreement expected to chance is recommended. Would it be possible to provide the kappa statistic?

   Response: we thank the reviewer for this suggestion. However, kappa statistic (Cohen's kappa, if this what reviewer means) is not appropriate in the case of our study since this statistical approach is designed to measure the agreement between qualitative (categorical) items. While distances that are investigated in our study are continuous (quantitative) variables. It is simply impossible to apply Cohen's kappa statistics to assess the agreement between distances estimated at the two visits, because the lengths can take any value and are not selected among a limited number of categories. In addition, we would like to alert the reviewer against using Cohen's kappa in general. For example, FDA Statistical Guidance on Reporting Results from Studies Evaluating Diagnostic Tests (https://www.fda.gov/media/71147/download) strongly advices against using Cohen's kappa statistics: "*One should also be aware that measures of overall agreement (including both overall percent agreement and Cohen's Kappa) can be misleading in this setting. In some situations, overall agreement can be good when either positive or negative percent agreement is very low. For this reason, FDA discourages the stand-alone use of measures of overall agreement to characterize the diagnostic performance of a test*". Another example from literature (*Pontius, Robert & Millones, Marco. (2011). Death to Kappa: Birth of quantity disagreement and allocation disagreement for accuracy assessment. International Journal of Remote Sensing. 32. 4407-4429. 10.1080/01431161.2011.552923.*) corroborates this conclusion: "*In the cases that we have seen, Kappa gives information that is redundant or misleading for practical decision making*". The examples are numerous and cannot all be cited here, but we hope that this can be helpful for the reviewer.

10. The authors mention low retest bias in line 179/180, however, the statistical analysis section does not describe how this was calculated, as the units of measurements do not correlate to the above statistical analysis section. Please clarify in the statistical analysis section how this was quantified and calculated.

    Response: we kindly disagree with the reviewer. Bias or systematic error is a very common term, and we don't think a description of how it is calculated is needed. While confidence interval or ICC are more advanced concepts, the details on how they are calculated are not required by the reviewer. Bias is defined and calculated as a mean difference between two measurements over the entire population. In our case, two measurements correspond to two visits in the hospital or to two home assessments with one week interval. Hence, the units of measurement are consistent with the definition.

11. Given the small sample size and concerns detected in the methods and statistical analysis section, the final conclusion in the discussion should be reworded. Instead of saying "strong evidence" in line 224, the authors could restate to suggest that FeetMe shows promising results for future exploration, rather than suggesting that the app can be used now.

    Response: we kindly disagree with the reviewer on his statement about the concerns regarding the methods and statistical analysis. We clarified the language to make these sections clearer and as we explain above, the reviewer's proposal regarding Cohen's kappa statistics is unapplicable to our data. We changed the language of the phrase so that it corresponds more precisely to our observations: "*Thus, at-home self-administered 6MWTs using the FeetMe technology were found to be at least, if not more, reliable than rater assessments conducted in a hospital setting, providing evidence that the FeetMe insoles could deliver an easy-to-use,*

reliable, and accurate solution allowing patients to conduct 6MWTs at home" (lines 232-235). We also updated the '*Conclusions'* section using a slightly more cautions language.

12. In line 175 of the results section, the authors describe a large difference between the FeetMe measurements and clinical measurements. Please provide additional information in the discussion section on why this may have occurred. For example, does this suggest that the app does not correlate well with gold standard clinical measurements, or does it require modifications in assessment?

    Response: the reviewer has misunderstood our statement. The large difference which we are referring to occurs between the measurements at the initial and the second hospital visit and this observation is just as valid for FeetMe, as it is for the rater. So, it does not suggest that the app does not correlate well with the gold standard, nor does it require modifications in assessment. Most likely, we see here the well-known learning effect [24]. To avoid such misunderstanding by the readers, we rewrote the sentence: "*The mean and SD of the 6MWDs measured at baseline were very similar to those estimated at Week 1 for the FeetMe evaluations conducted at home. On the contrary, when comparing the measurements taken during the initial hospital visit with those of the second visit, we noted a notable disparity in both the mean and SD estimators (Table 1). This observation held true for both the rater assessments and the FeetMe assessments*" (lines 196-200). We also mentioned the learning effect in the discussion: "*Furthermore, the learning effect [24], which was evident in the in-hospital measurements, was not observed in the measurements conducted at home. This difference suggests that the FeetMe insoles facilitate consistent and unbiased assessments during at-home testing, unaffected by the learning curve observed in the hospital setting*" (lines 229-232).

13. When describing the use of new technology in research, it is essential to describe any potential ethical concerns. The authors mention that technical details of the app have already been published, however, it is important to note where the data is stored, for how long it is stored, who has access to the data, and how healthcare professionals access the information. Please include a section discussing the potential ethical concerns of the app.
    Response: we thank the reviewer for their remark. It is essential to be transparent about how data is handled and provide clarity to readers about the security measures in place. We detailed this in the manuscript: "*The data then were automatically sent to a medical service-certified secure server and test results could be displayed in the application or on the associated web platform, the FeetMe Mobility Dashboard. The access to the data and to the test results is fully restricted to authorized persons only*" (lines 130-133). Considering the potential ethical considerations, we acknowledge your viewpoint. Nevertheless, it is essential to highlight that the proposed solution aligns with the current regulations in force, rendering a separate, dedicated section on ethical concerns possibly redundant. Due to the limited available material, it might not be feasible to create a comprehensive segment solely focused on ethical aspects.

REVIEWER 3 COMMENTS:

We extend our gratitude to the reviewer for the constructive discussion, and we believe that these revisions will enhance the clarity and accuracy of our findings. Further down, we delve into our responses to the comments provided by the reviewer.

1. Structure and flow. A large part of the discussion feels like it is providing the justification for this research and therefore should set the scene for the research within the introduction (lines 227-253)

   Response: we thank the reviewer for this observation. However, we found only one such paragraph, that we moved to the '*Introduction*' section (lines 64-85). As for the rest of the '*Discussion*' section, it either exposes our findings or analyses previously published results with the goal of comparing these with our findings. Even though we found that some of the reported results can't be directly compared with ours, we believe that their description still belongs to the '*Discussion*' section.

2. The figure 2 and figure 3 should be moved below the commentary, which explains the figure which follows next (i.e. move lines 158-160 above figure 2 and move figure 3 below lines 167-171).

   Response: we thank the reviewer for this proposal. We moved the figures as good as possible in the suitable areas of the manuscript, taking into account the constraints of the journal layout and the presence of Table 1 next to figures.

3. I suggest a section title for the statistical analyses, provides a clear distinction from the population demographics, which starts with a short introduction to the statistical analyses section.

   Response: we thank the reviewer for this suggestion. To remediate this issue, we moved several paragraphs from the '*Demographics and population distribution*' to newly created section to '*Results of the test*' section (lines 183-194). The '*Demographics and population distribution*' section now only contains the relevant information.

4. Table 1 should be moved lower down the section, where the results within it are described

   Response: we thank the reviewer for this proposal. We moved Table 1 as close as possible to the paragraph in which it is first mentioned.

5. Results interpretation, the researchers have highlighted and explained the difference between hospital rater and the FeetMe at home tests, however, have not sufficiently explained or interpreted the results between the FeetMe at home and in hospital results. A further commentary outlining the differences in the results and exploring possible reasons for this should be expanded upon.

   Response: we appreciate the reviewer's valuable feedback and the opportunity for discussion. We would like to clarify our position and address the points raised. First, we would like to emphasize that the primary focus of our report was on comparing the test-retest reliability of different measurement methods and conditions, as reflected by various statistical indicators such as the ICC, bias, and limits of agreement (presented in Table 2) and not on comparing individual measurements described by the statistical indicators such as mean distance or its SD at baseline or at Week 1 (shown on Table 1). Second, we would like to highlight the similarity between the reliabilities of in-hospital measurements conducted by the rater and those by FeetMe, as indicated in Table 2, which convinced us in our interpretation to consider the differences between at-home and in-hospital testing conditions, rather than to solely compare the measurement methods by themselves. During in-hospital testing the presence of the rater watching the participant and giving them instructions and the time remaining until the end of the test apparently may influence the results to a greater extent than the choice of

measurement method, as our data shows. We have taken the reviewer's point into account and have revised the manuscript to clarify this particularity of our comparative approach and we explicitly highlighted the learning effect [24] observed for in-hospital measurements (both by the rater and by FeetMe) but not for at-home measurements, as elaborated in the '*Discussion*' section.

6. Clearly defined implications for future research (or similar) and learnings from this study sections, would add value to the manuscript as would a section more clearly defined as limitations.

   Response: we thank the reviewer for this proposal. We added '*Study limitations*' section. As for the implications for future research, we believe that in the new revision they are clearly mentioned, even though they are not all concentrated in a single paragraph.

7. Overall improvements could be made to structure and flow with use of headings to break up longer sections to aid readability.

   Response: we thank the reviewer for this proposal. We split the manuscript into additional sections with headings '*Inclusion*', '*Results of the test*', '*Study limitations*'.

We greatly appreciate your input and inquiries, which have allowed us to enhance our manuscript. We have diligently integrated your feedback and are optimistic that these revisions will encourage you to consider accepting our submission.

Sincerely,

Andrey Mostovov

Chief Technology Officer

FeetMe SAS

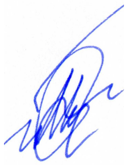

Supplement: Attachment — Submitted filename: 230816-rebuttalLetter_PLOS.pdf [file pdig.0000262.s001.pdf]
