# Supplementary material for: Test-retest reliability of the six-minute walking distance measurements using FeetMe insoles by completely unassisted healthy adults in their homes
Source: PLOS Digit Health. 2023 Nov 22;2(11):e0000262. doi: 10.1371/journal.pdig.0000262 (PMC10664940; doi:10.1371/journal.pdig.0000262)

Haleh Ayatollahi, PhD
Section Editor
PLOS Digital Health

September 29th, 2023.

Dear Dr. Ayatollahi,

I trust this letter finds you well. We extend our sincere appreciation for the acceptance of our manuscript and are truly honored to be able to contribute to the scientific discourse within the pages of your distinguished journal. To align with your journal's standards and resolve the remaining comments and question we updated our manuscript that we are resubmitting to you. To facilitate your review of this updated version, we have prepared a detailed response addressing each of the questions and comments from your letter dated September 12th, 2023.

EDITOR REQUESTS:

We thank the editor team for their remarks and suggestions which we have thoroughly addressed, as elaborated in the following section.

1. Please review your reference list to ensure that it is complete and correct. If you have cited papers that have been retracted, please include the rationale for doing so in the manuscript text, or remove these references and replace them with relevant current references. Any changes to the reference list should be mentioned in the rebuttal letter that accompanies your revised manuscript. If you need to cite a retracted article, indicate the article's retracted status in the References list and also include a citation and full reference for the retraction notice.

   Response: we verified the reference list and confirm that it is complete and correct. We put the reference of the work that was not yet published at the time of our previous submission and that is now published (reference 20, line 98). The manuscript does not contain any citations of unpublished works.

2. We do not publish any copyright or trademark symbols that usually accompany proprietary names, eg ©, ®, ™ (e.g. next to drug or reagent names). Please remove all instances of trademark/copyright symbols throughout the text, including ® on page 17.

   Response: we apologize for forgetting to remove the symbol in this occurrence, now it is solved.

3. The title is a bit long. Could you please make it shorter?

   Response: we changed the title to "*Test-retest reliability of the six-minute walking distance measurements using FeetMe insoles by completely unassisted healthy adults in their homes*".

4. Please follow the journal instructions for the manuscript preparation including the abstract format.

   Response: thank you for noting this item. We updated the abstract and the format of the refence list to match your journal requirement. We are confident that our manuscript now follows them entirely.

5. Please add appropriate keywords based on the MeSH terms.

   Response: appropriate keywords based on the MeSH terms were added as per your request.

6. Please ensure that the aim of the study is the same in the abstract, introduction, etc.

   Response: we updated the abstract, the author's summary, introduction, discussion and study limitations section to make them as consistent as possible. Please note that we use the terms test-retest reliability and repeatability alternatively as they are synonyms.

REVIEWER 1 COMMENTS:

We would like to convey our appreciation to the reviewer for their time and for offering us valuable feedback. In the following section, we present our responses to the reviewer's comments.

1. While the authors have specified the CE/FDA classifications regarding the use of the application, the data handling policies were not specified. Data collection is explicitly mentioned in line 125 where the relevant data handling policy that was adhered to can be specified as good practice for scientific publication.

   Response: we thank the reviewer for this suggestion, we added the following description (lines 136 – 138): "*The data was handled in accordance with the French data protection authority's (CNIL) reference methodology MR-003 as well as in accordance with the requirements of the European Union General Data Protection Regulation (GDPR)*".

2. Regarding the title, considering the limited number of interventions, lack of control groups and the focus on healthy participants (while aiming to address patients with gait issues), it would be advisable to rephrase the study title as a feasibility assessment or pilot study to be more indicative of the study scope.

   Response: we understand the reviewer's concern, we changed the title to "*Test-retest reliability of the six-minute walking distance measurements using FeetMe insoles by completely unassisted healthy adults in their homes*".

We sincerely value your input and inquiries, which have played a crucial role in the completion of our manuscript. We have thoroughly incorporated your feedback and are confident that these revisions have prepared the manuscript for publication.

Sincerely,

Andrey Mostovov

Chief Technology Officer

FeetMe SAS

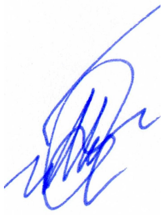

Supplement: Attachment — Submitted filename: 230921-rebuttalLetter_PLOS.pdf [file pdig.0000262.s002.pdf]
